# The role of type I collagen in white matter injury

**DOI:** 10.1007/s12565-025-00881-y

**Published:** 2025-07-18

**Authors:** Reiji Yamazaki, Nobuhiko Ohno

**Affiliations:** 1 Department of Anatomy, Division of Histology and Cell Biology, School of Medicine, Jichi Medical University, Shimotsuke, Japan; 2 Division of Ultrastructural Research, National Institute for Physiological Sciences, Okazaki, Japan

**Keywords:** Remyelination, Oligodendrocyte, Type I collagen, White matter injury

## Abstract

White matter injury is a cerebral pathology marked by the loss of oligodendrocytes and the resultant demyelination. Various mechanisms induce white matter injury, including ischemic stroke and multiple sclerosis. Oligodendrocytes regenerate white matter in the central nervous system in a process called remyelination, ensheathing demyelinated axons with new myelin. While failures of remyelination can be observed in progressive multiple sclerosis and after ischemic stroke, the mechanisms of impaired white matter regeneration remain unclear. In this review, we primarily focus on our recent work while summarizing studies reporting on mouse models of internal capsule demyelination and discuss inhibitory factors affecting white matter regeneration. In addition, we provide recent findings on the role of type I collagen as an inhibitory molecule of remyelination in white matter lesions.

## Introduction

The myelin sheath of nerves in the central nervous system (CNS) is formed by specialized glial cells called oligodendrocytes (Bercury and Macklin 2015, Demmings et al. 2025). Myelin acts as an insulator and is essential for saltatory conduction (Angulo 2025; Nave and Werner 2014). Oligodendrocytes have multiple processes and generate myelin, which surrounds axons (Chang et al. 2016; Yamazaki and Ohno 2024b; Yu et al. 2023). Brain regions containing numerous myelinated fibers are referred to as white matter due to their macroscopic appearance resulting from the lipid-rich structure of myelin (Fields 2010; Zavolskova et al. 2025). White matter damage is observed in various neurological diseases, such as multiple sclerosis (MS), traumatic brain injury (TBI), and after ischemic stroke (Armstrong et al. 2024; Braun et al. 2017; Compston and Coles 2008; Reich et al. 2018; Wang et al. 2016; Youssef et al. 2021). This white matter injury is associated with demyelination and can contribute to motor and sensory paralysis, cognitive dysfunction, depression, and dementia (Compston and Coles 2008; Reich et al. 2018; Wang et al. 2016; Carmichael and Llorente 2023). Regeneration of white matter through the process of remyelination is therefore crucial for functional recovery (Youssef et al. 2021; Huang et al. 2023; Yamazaki et al. 2021). Recently, various therapeutic approaches have been investigated to address diseases involving white matter damage. From the perspective of regenerative medicine, cell therapy has attracted significant interest. It has been suggested that the transplantation of neural stem cells can induce myelin regeneration after damage, including from MS and spinal cord injury (Li et al. 2024; Scolding et al. 2017). While a variety of regeneration inhibitors and signaling pathways have been identified (Keough et al. 2016; Xie and Zheng 2008; Ghorbani and Yong 2021), there are currently no developed therapies for the promotion of remyelination and white matter repair.

Several animal models are widely used in basic research on white matter injury to elucidate pathological mechanisms and evaluate drug efficacy (Constantinescu et al. 2011; Matsushima and Morell 2001; Robinson et al. 2014; Yamazaki and Ohno 2024a). However, conventional animal models offer limited control over the extent of demyelination and resulting neurological symptoms. Consequently, producing reproducible evaluations of motor deficits and tissue regeneration associated with white matter injury has been a challenge in these models. Previously, we developed a novel mouse model of demyelination by injection of the detergent lysolecithin (LPC) into the internal capsule (IC) (Yamazaki et al. 2021). LPC can induce demyelination through membrane-dissolving action and can be injected directly into myelinated fibers to generate a model of white matter injury (Blakemore and Franklin 2008; Keough et al. 2015). This IC demyelination mouse model induces acute motor deficits and subsequent remyelination-associated functional recovery. Therefore, the model can be used to assess both remyelination and functional recovery after drug treatment (Yamazaki et al. 2023). During the development of this demyelination mouse model, we compared it to a white matter infarction model in which the vasoconstrictor endothelin-1 (ET1) was injected into the IC. We observed that type I collagen deposits were a common factor in white matter areas that did not regenerate (Yamazaki et al. 2021; Yamazaki et al., 2025). Previous analyses of the brains of human patients with MS have indicated elevated gene expression of several collagen types, including *Col1a1, Col3a1, Col5a1,* and *Col5a2* in lesional compared with non-lesional areas (Mohan et al. 2010; van Horssen et al. 2007). Recently, it was reported that MS lesions are characterized by the deposition of type I collagen fibers in the perivascular spaces of small- and medium-sized veins (Absinta et al. 2019). A study of oligodendrocyte cultures found that their differentiation into mature oligodendrocytes was prevented in the presence of fibroblasts, the primary collagen-forming cells (Yahn et al. 2020). Considering these findings, we hypothesized that type I collagen inhibits white matter regeneration. However, its role in demyelination was unclear, leading us to investigate its potential contribution to white matter lesions.

In this review, we propose a recovery-inhibiting role for type I collagen secreted in white matter lesions, based on our recent work.

## Type I collagen deposition in white matter lesions

Type I collagen is the most abundant collagen in the human body (Naomi et al. 2021; Rossert and de Crombrugghe 2002). The *Col1a1* gene encodes the alpha-1 chain of type I collagen (Naomi et al. 2021; Rossert and de Crombrugghe 2002). Collagen fibers are the major structural proteins in the body, formed by the aggregation of collagen molecules and repeated cross-linking reactions (Fratzl 2008; Nimni and Harkness 2018). As a major component of the extracellular matrix, these fibers play a role in maintaining strength and elasticity, particularly in connective tissues, such as skin, bones, tendons, and cartilage (Fratzl 2008; Nimni and Harkness 2018). However, disease or injury can cause fibrosis, a condition in which collagen and other extracellular matrix proteins are produced in excess and subsequently deposited in tissues (Henderson et al. 2020; Wynn 2008). The major collagen-producing cells are fibroblasts, which are not present in the brain parenchyma but are localized around the meninges in the CNS.

**Fig. 1:**
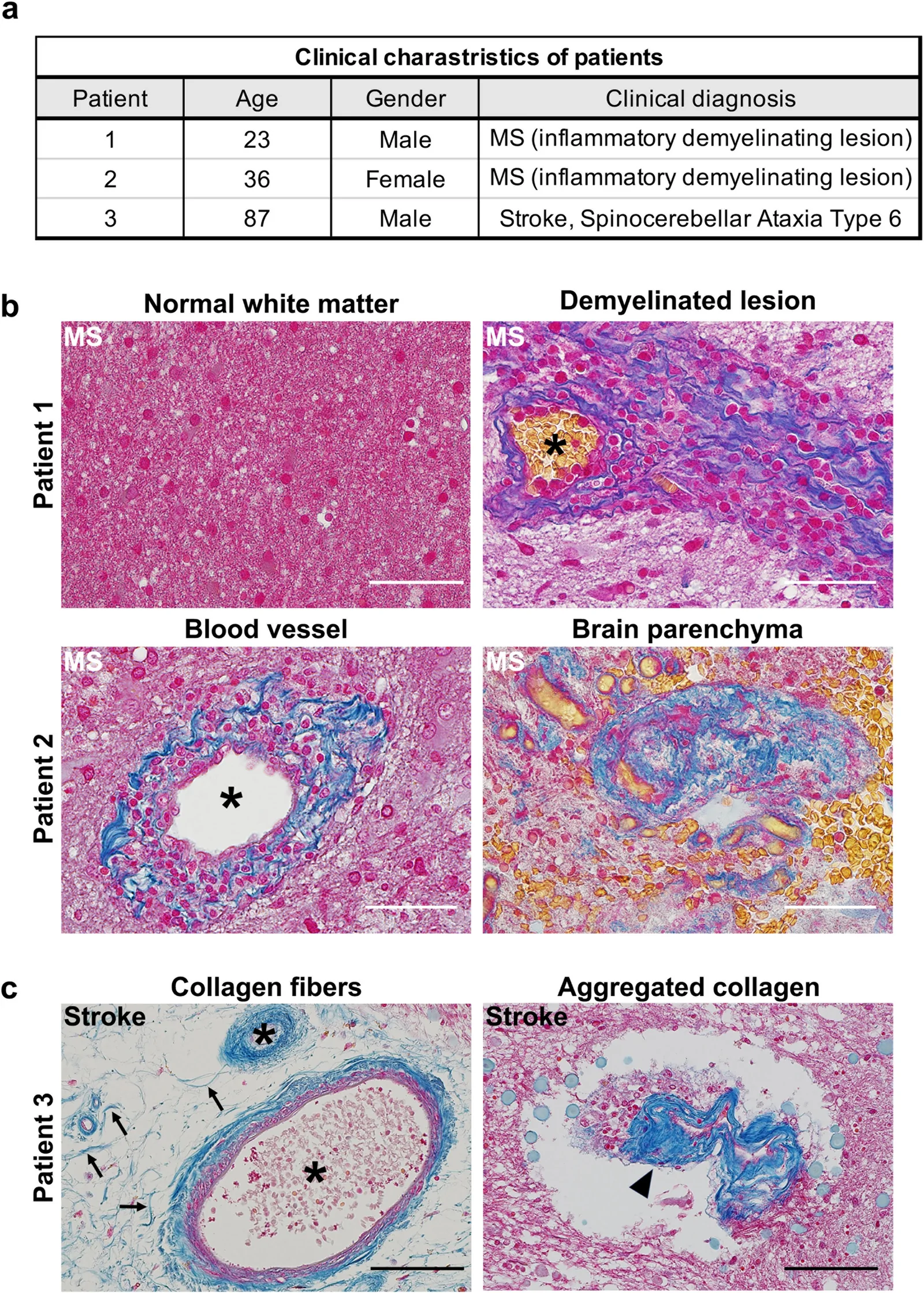
Histological analysis of white matter lesions in human brain tissue. **a** Clinical characteristics of multiple sclerosis (MS) and stroke patients.** b** Azan staining of paraffin-embedded sections from MS patients. Collagen fibers were not observed in normal-appearing white matter of MS Patient 1. Collagen fibers were detected around blood vessels and brain parenchyma in the white matter lesions of all MS patients. Collagen fibers present in the white matter lesions of a patient diagnosed with MS. Scale bars, 50 µm. **c** Azan staining of paraffin-embedded sections from a stroke patient. Collagen fibers (left panel, black arrows) and aggregated collagen (right panel, black arrowhead) were observed in the white matter lesions of a stroke patient. Scale bars, 100 µm. Asterisks indicate collagen-positive blood vessels

In our recent study (Yamazaki et al. 2025), we obtained paraffin-embedded sections of human brain samples from MS and stroke patients through the Japan Brain Bank Net (JBBN) (Fig. 1a). Biopsy tissue from MS patients and autopsy tissue from stroke patients were examined for collagen fiber deposition in white matter lesions by Azan staining (Fig. 1b, c). Azan staining is a histological technique for detecting collagen fibers (Fig. 1, blue). In the white matter lesions of MS patients, we observed large amounts of collagen around blood vessels (Fig. 1b, asterisks) and in the brain parenchyma away from blood vessels (Fig. 1b, Patient 2, right panel). In contrast, there was no collagen in normal-appearing white matter (Fig. 1b, Patient 1, left panel). Similarly, secreted-collagen fibers (Fig. 1c, black arrows), collagen fibers around blood vessels (Fig. 1c, asterisks), and aggregated collagen (Fig. 1c, black arrowhead) were all observed in areas of white matter damage in the autopsy tissue of stroke patients (Fig. 1c, Patient 3). TBI is also a major cause of white matter injury. Changes in the extracellular matrix following TBI have been reported to play an important role in its pathophysiology, controlling inflammation, blood–brain barrier function, and regeneration (Aqel et al. 2023; George and Geller 2018; Kursancew et al. 2024). Previously, collagen matrix implantation was shown to reduce lesion volume and neuronal loss and improve cognitive dysfunction in a rat model of TBI (Shin et al. 2015). In a similar model, the transplantation of collagen scaffolds, incorporating either soluble Nogo receptor or human marrow stromal cells, improved spatial learning and sensorimotor function and reduced lesion volume (Elias and Spector 2015; Lu et al. 2007). However, there are no reports on the role of type I collagen deposited in white matter lesions in TBI. Therefore, histological analysis of the white matter of TBI patients is an important future research direction.

**Fig. 2:**
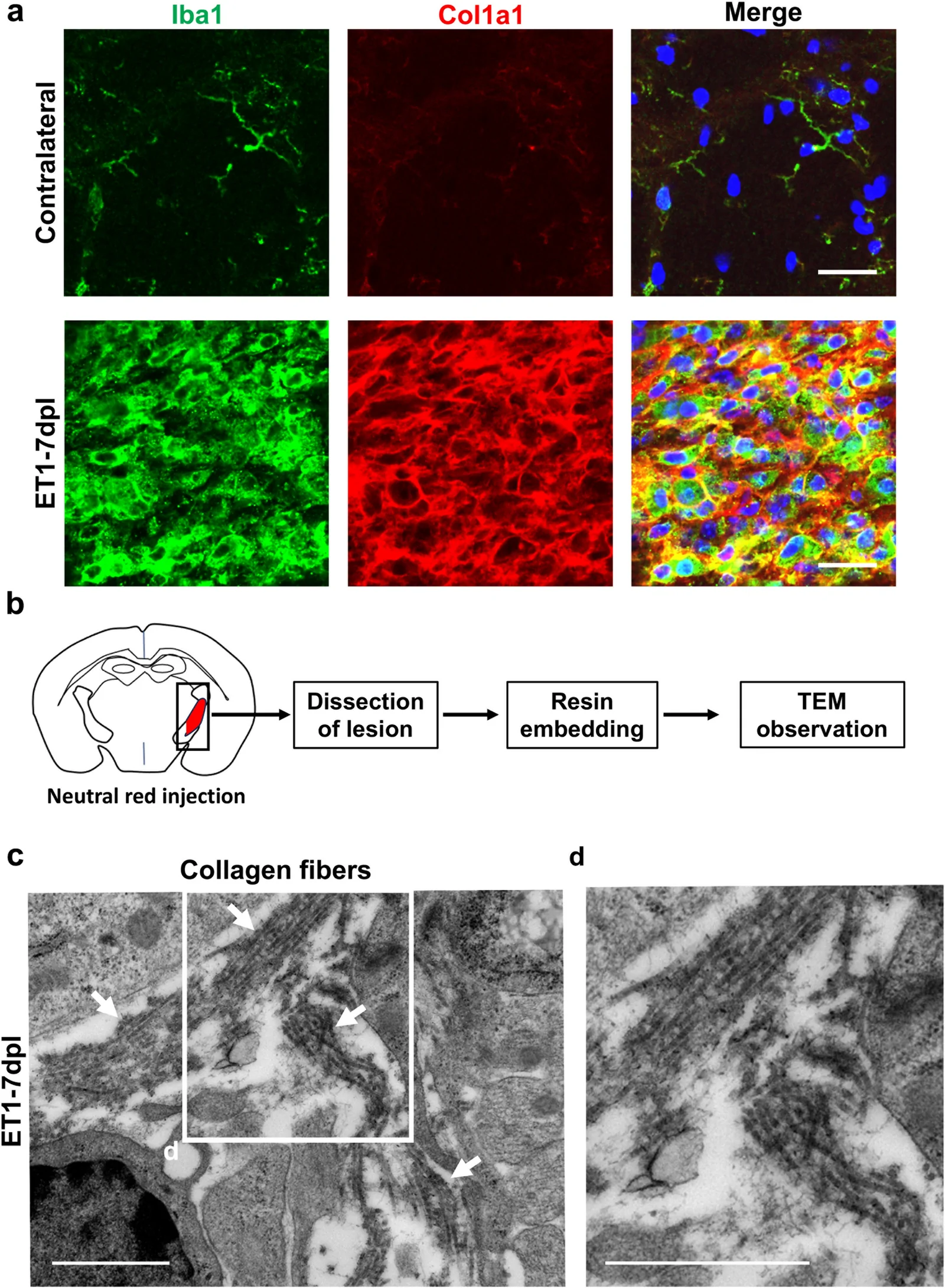
Histological analysis of endothelin-1 (ET1)-induced white matter lesions of the internal capsule (IC) in mice. **a** Double immunohistochemical analysis of coronal sections of ET1-induced IC at 7 days post-lesion (dpl) and contralateral IC at 7 dpl using anti-Iba1 (green) and anti-Col1a1 (red) antibodies. Nuclei were counterstained with Hoechst (blue). Scale bars, 20 µm. **b** Experimental design of transmission electron microscopy (TEM) observation using neutral red (NR) labeling. Mice were treated with NR by intraperitoneal injection. NR-labeled tissue was dissected at 7 dpl. The tissue was embedded in epoxy resin. Ultrathin sections were prepared and analyzed by TEM. **c** TEM analysis of ET1-induced lesioned IC at 7 dpl. Collagen fibers are present in the white matter lesion (white arrows). Scale bar, 1µm. **d** An enlarged image of the white boxed area in **c**. Scale bar, 1 µm

We also examined whether collagen fibers were secreted in the white matter lesions of an animal model. We generated white matter lesions by injecting ET1 into the IC. ET1-induced white matter injury prevents regeneration (Blasi et al. 2015; Yamazaki et al. 2021). Microglia and macrophages migrate to lesions after CNS injury (Mayer and Fischer 2024; Strogulski et al. 2023). Immunofluorescence staining was performed using an anti-Iba1 antibody as a microglia/macrophage marker and an anti-Col1a1 antibody to detect type I collagen in the lesions (Fig. 2a). No Col1a1 signal was detected in the contralateral IC. In contrast, colocalized Col1a1-positive and Iba1-positive signals were detected in white matter lesions at 7 days post-lesion (dpl) after ET1 injection (Fig. 2a). Electron microscopy (EM) can be used to visualize the fibrous structure of collagen. We applied neutral red (NR) labeling, a method of observing white matter lesions, for EM analysis (Yamazaki and Ohno 2024c). After white matter injury at 7 dpl, NR was injected to allow macroscopic observation of the lesions. NR-labeled tissue was dissected, embedded in resin, and observed by transmission EM (TEM) (Fig. 2b). Notably, secreted-collagen fibers were observed in the extracellular space after white matter injury (Fig. 2c, d).

Meanwhile, abnormal collagen fiber-like structures were observed in the lysosomes of microglia- and macrophage-like cells in the chronic phase following white matter injury (Yamazaki et al. 2025). Collagen fibers are generally degraded by phagocytosis (Adachi et al. 1997; Ross et al. 2003). The phagocytosis of myelin debris by microglia and macrophages at lesion sites is essential for white matter repair (Jia et al. 2023; Yu et al. 2022; Miron 2017). These results indicate that collagen fibers were secreted in white matter lesions and subsequently phagocytosed by microglia- and macrophage-like cells (Yamazaki et al. 2025).

## Type I collagen-producing cells in white matter lesions

Next, to identify type I collagen-producing cells, we performed a combination of immunohistochemistry and *in situ* hybridization at 7 dpl after ET1 injection. We found that of all the Iba1-positive cells accumulating in the lesion area, approximately 20% were Col1a1-positive cells (Yamazaki et al. 2025). Iba1-positive cells in the lesions included the intrinsic microglia and monocyte-derived macrophages (MDMs) that influx from the periphery after brain injury (Hou et al. 2023; Baaklini et al. 2019). Previous studies have reported that while MDMs infiltrating lesions after blood–brain barrier disruption are primarily protective, they can also exacerbate disease through the secretion of inflammatory cytokines (Baaklini et al. 2019; Montilla et al. 2023; Wicks et al. 2022). Although MDMs have a gene expression pattern similar to that of microglia, they can be distinguished by their expression of the C–C chemokine receptor (CCR2) (Ajoolabady et al. 2024; Kim and Cho 2016; Yamasaki et al. 2014). Furthermore, MDMs are classified as either pro-inflammatory or anti-inflammatory. Inflammatory MDMs secrete pro-inflammatory factors, including TNFα, IL-1β, IL-6, IFNγ, reactive oxygen species (ROS), and nitric oxide (NO) (Mildner et al. 2009; Shemer and Jung 2015). In contrast, anti-inflammatory MDMs are characterized by the expression of Arginase-1 and YM1 and secrete the anti-inflammatory cytokines IL-4 and IL-10 (Butovsky et al. 2006; Quarta et al. 2021). Recently, macrophage diversity has been reported across various diseases, suggesting that perivascular macrophages may contribute to cerebral arteriolosclerosis through the secretion of collagen fibers (Zhan et al. 2025; Inagaki et al. 2021).

We then hypothesized that MDMs secrete type I collagen and proceeded with our analysis. The results indicated that most *Col1a1* mRNA-positive cells in white matter lesions were MDMs (Yamazaki et al. 2025). Subsequent EM analysis revealed that cells with monocyte-like morphology secreted-collagen fibers (Yamazaki et al. 2025). These results suggest that the collagen fibers observed in areas of white matter damage are produced by MDMs invading from the periphery.

## Inhibition of white matter regeneration by type I collagen

Previous studies have identified various factors that inhibit remyelination. These include Fyn–RhoA–ROCK, BMP, and Wnt/Notch signaling pathways, as well as the molecules Sema3A and Lingo-1 (Baer et al. 2009; Fancy et al. 2009; Mi et al. 2007; Sabo et al. 2011; Syed et al. 2011). These signaling pathways and molecules prevent remyelination by directly or indirectly affecting oligodendrocytes, and are considered potential therapeutic targets. Recently, clemastine, an antagonist of the M1 muscarinic acetylcholine receptor in oligodendrocyte precursor cells, was identified by high-throughput screening platforms using micropillar arrays as a drug candidate for the promotion of remyelination (Mei et al. 2014, 2016; Yamazaki and Ohno 2025). Meanwhile, extracellular matrix molecules, including chondroitin sulfate proteoglycans (CSPGs) and hyaluronan, are also reported to block remyelination (Ghorbani and Yong 2021; Keough et al. 2016). CSPGs and hyaluronan accumulate in demyelinated lesions (Back et al. 2005; Sobel and Ahmed 2001). Previous studies have reported on both the inhibition of extracellular matrix production and its promotion through degradation. Several therapeutic candidate compounds targeting CSPG have been identified. Of these, fluorosamine, 2-arachidonoylglycerol (2-AG), and xyloside have been administered to models of demyelination and spinal cord injury, resulting in a reduction in CSPG accumulation and promotion of OPC differentiation (Feliú et al. 2017; Keough et al. 2016; Lau et al. 2012; Pendleton et al. 2013; Siebert and Osterhout 2011). Downstream signaling of CSPGs has also been identified. CSPGs can interact with receptors, including protein tyrosine phosphatase sigma (PTPσ), leukocyte common antigen (LAR), and Nogo receptor (NgR1/3) (Fisher et al. 2011, Lang et al. 2015; Sharma et al. 2012; Shen et al. 2009). Inhibition of these signals promotes recovery in some animal models, including EAE, LPC-induced demyelination, and spinal cord injury (Lang et al. 2015; Luo et al. 2018; Shen et al. 2009). Therefore, the extracellular matrix is a major factor in inhibiting remyelination.

Building on our findings and previous reports, we sought to clarify whether type I collagen also inhibits remyelination. We assessed motor functional recovery, oligodendrocyte differentiation, and remyelination after injecting a solution of LPC mixed with type I collagen (LPC/collagen) into the IC. Motor functional recovery was prevented in LPC/collagen-injected mice (Yamazaki et al. 2025). In contrast, control mice treated with LPC/collagen co-injected with collagenase type I—which degrades type I collagen—exhibited recovered motor function (Yamazaki et al. 2025). Type I collagen exacerbated neuroinflammation by activating microglia and inducing astrogliosis. Finally, we demonstrated that oligodendrocyte differentiation and remyelination were inhibited in the presence of type I collagen after demyelination (Yamazaki et al. 2025). Taken together, these findings suggest that type I collagen is an inhibitory factor for white matter regeneration.

## Conclusion

**Fig. 3:**
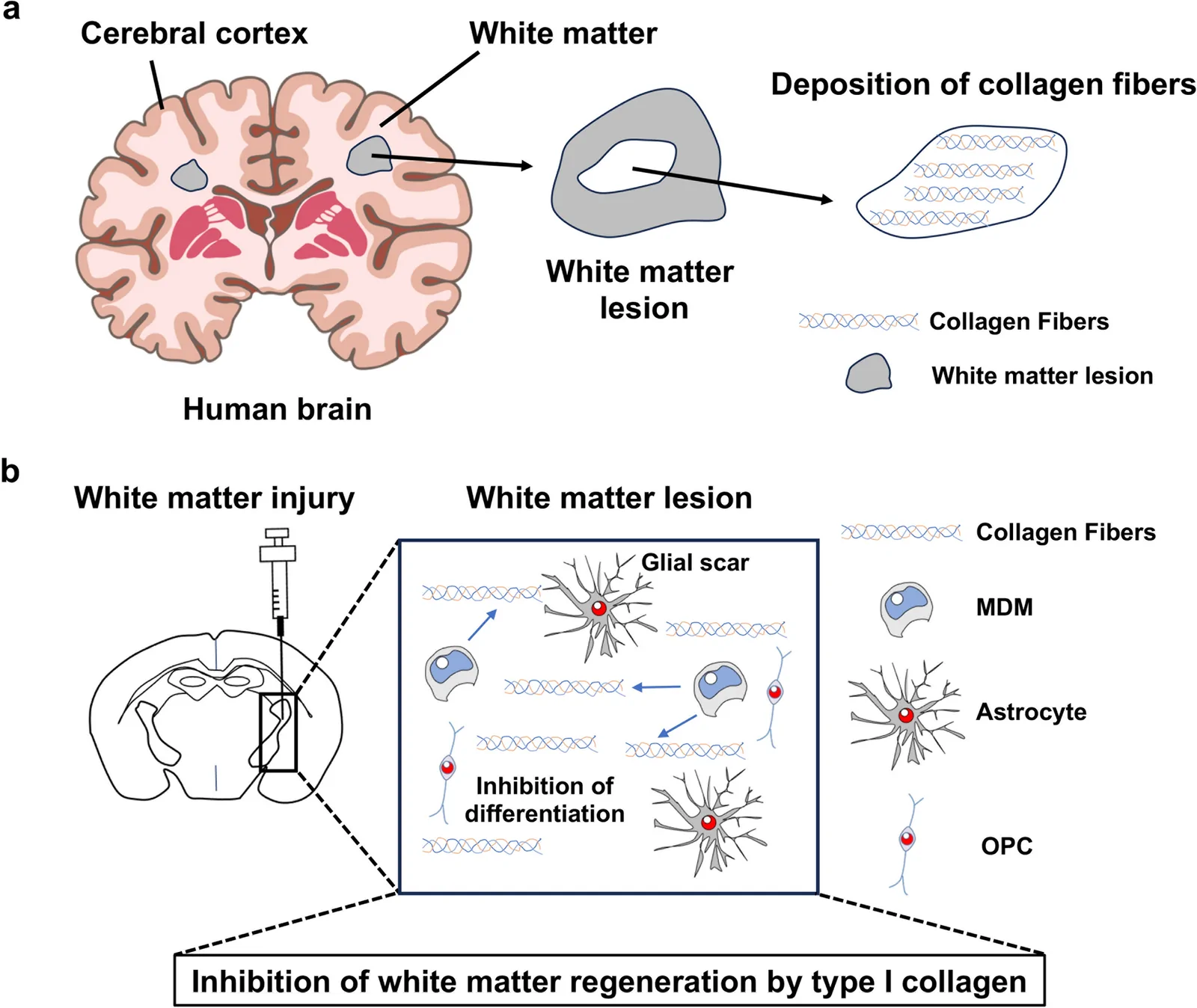
Summary of this review. **a** White matter lesions are observed in multiple sclerosis and stroke patients. Secreted collagen fibers and aggregated collagen are deposited in some white matter lesions in human brain tissue. **b** White matter injury is induced by injection of endothelin-1 into the IC. In the white matter lesion, collagen fibers are produced by monocyte-derived macrophages (MDM). Secreted collagen fibers prevent the differentiation of oligodendrocyte precursor cells (OPCs) and promote glial scar formation by astrocytes in the white matter lesion. White matter regeneration is impaired by type I collagen

Previous postmortem analyses of the brains of MS patients have only detected collagen around blood vessels (Absinta et al. 2019). However, our study also found lesions with high levels of collagen production present in parenchymal brain regions distal from blood vessels in biopsy brain tissue from young MS patients with active demyelinating lesions (Fig. 3a). The role of MDMs in demyelinated lesions is complicated, with both protective functions and exacerbation of inflammation reported across various pathological conditions (Quarta et al. 2021; Wicks et al. 2022). We have demonstrated that MDMs produce type I collagen within white matter lesions (Fig. 3b). Furthermore, our findings suggest that type I collagen secreted in white matter lesions prevents white matter regeneration and functional recovery (Fig. 3b). The question of whether type I collagen directly or indirectly inhibits oligodendrocyte differentiation has not been fully clarified by our studies. The mechanism by which type I collagen mediates the inhibition of remyelination is unclear. However, type I collagen-related signaling may act upstream of Fyn–RhoA–ROCK, BMP, Wnt/Notch, and other unknown inhibitory pathways associated with CNS remyelination. Further clarifying the detailed mechanism by which type I collagen inhibits regeneration could establish it as a novel therapeutic target for white matter injury.

## Data Availability

All relevant data are included within this manuscript. Raw data will be made available on request by qualified researchers.
